# Supplementary material for: Monolithic Integration of Semi-Transparent and Flexible Integrated Image Sensor Array with a-IGZO Thin-Film Transistors (TFTs) and p-i-n Hydrogenated Amorphous Silicon Photodiodes
Source: Nanomaterials (Basel). 2023 Oct 31;13(21):2886. doi: 10.3390/nano13212886 (PMC10648663; doi:10.3390/nano13212886)
Supplement: Supplementary file 1 [file nanomaterials-13-02886-s001.zip › nanomaterials-2631019-supplementary.pdf]

Supporting Information

Monolithic Integration of Semi-Transparent and Flexible Integrated Image Sensor Array with a-IGZO Thin-Film Transistors (TFTs) and p-i-n a-Si:H Photodiodes

Donghyeong Choi [1,2†], Ji-Woo Seo [1,2†], Jongwon Yoon[1], Seung Min Yu[3], Jung-Dae Kwon[1], Seoung-Ki Lee[2]*, and Yonghun Kim[1]*

[1]Department of Energy and Electronic Materials, Surface Nano Materials Division, Korea Institute of Materials Science (KIMS), 797 Changwondaero, Sungsan-gu, Changwon, Gyongnam 51508, Republic of Korea; baru2300@kims.re.kr (D.C.); wldn0816@kims.re.kr (J.S.); jwyoon@kims.re.kr (J.Y.); jdkwon@kims.re.kr (J.K.); kyhun09@kims.re.kr (Y.K.)

[2]School of Materials Science and Engineering, Pusan National University, 2 Busandaehah-ro 63-beon-gil, Geumjeng-gu, Busan 46241, Republic of Korea; ifriend@pusan.ac.kr (S.L.)

[3]Analytical Research Division, Korea Basic Science Institute, Jeonju 54907, Republic of Korea; smyu040 9@kbsi.re.kr; smyu0409@kbsi.re.kr (S.M.Y.)

*****Co-correspondence: ifriend@pusan.ac.kr (S.L.); kyhun09@kims.re.kr (Y.K.)

Received: date; Accepted: date; Published: date

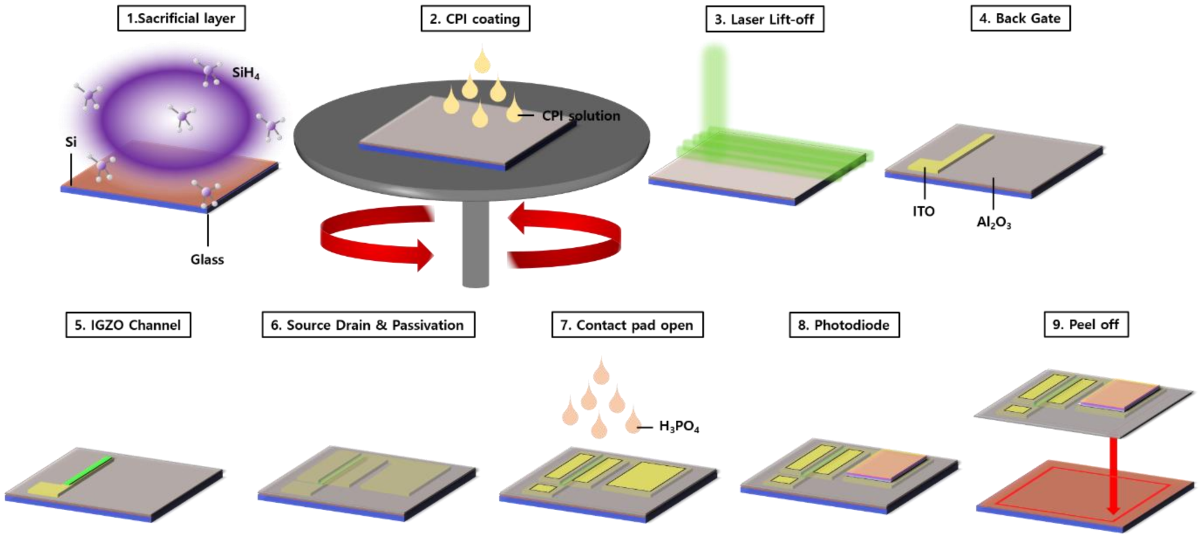

Figure S1. Detailed schematic fabrication process for a 1T-1D integrated image sensor of flexible a-IGZO TFT / a-Si:H photodiode on CPI film

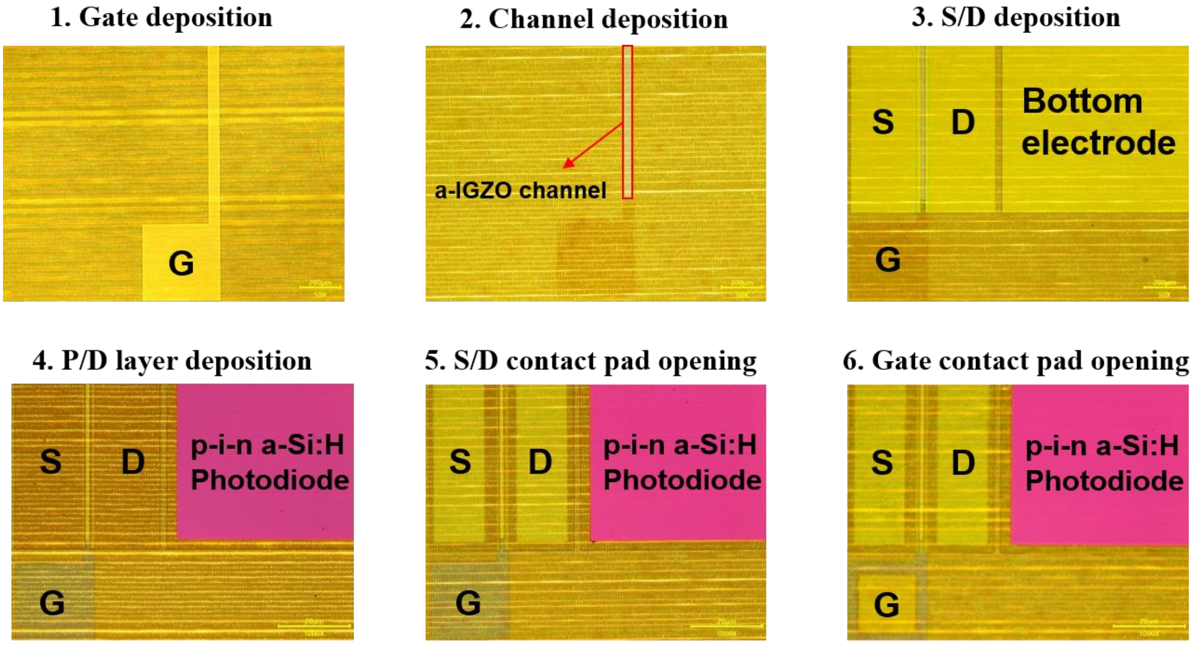

Figure S2. OM image illustrating the fabrication process of a 1T-1D structured image sensor.

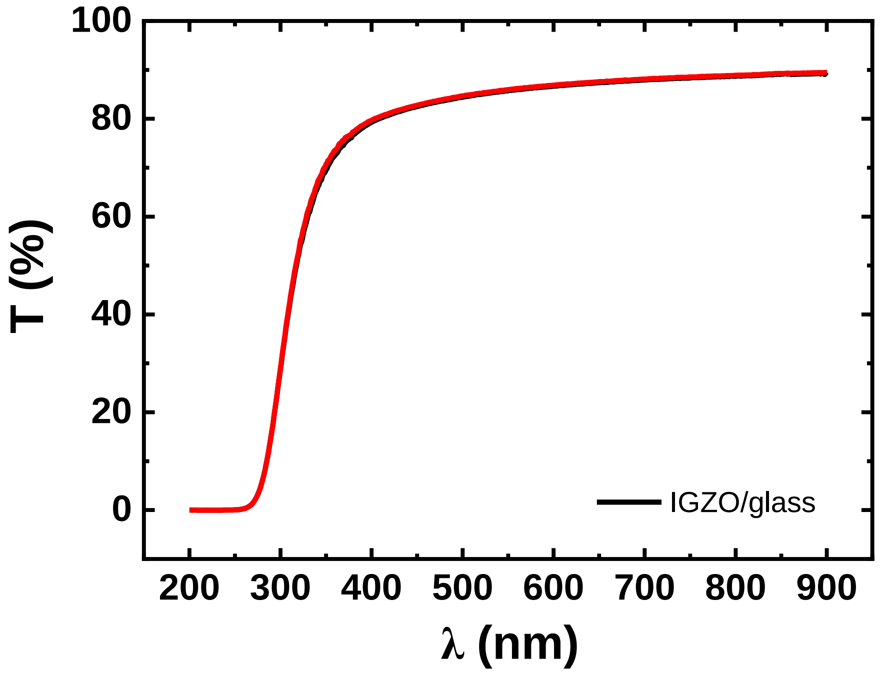

Figure S3. Transmittance spectra of the a-IGZO thin film on the glass substrate.

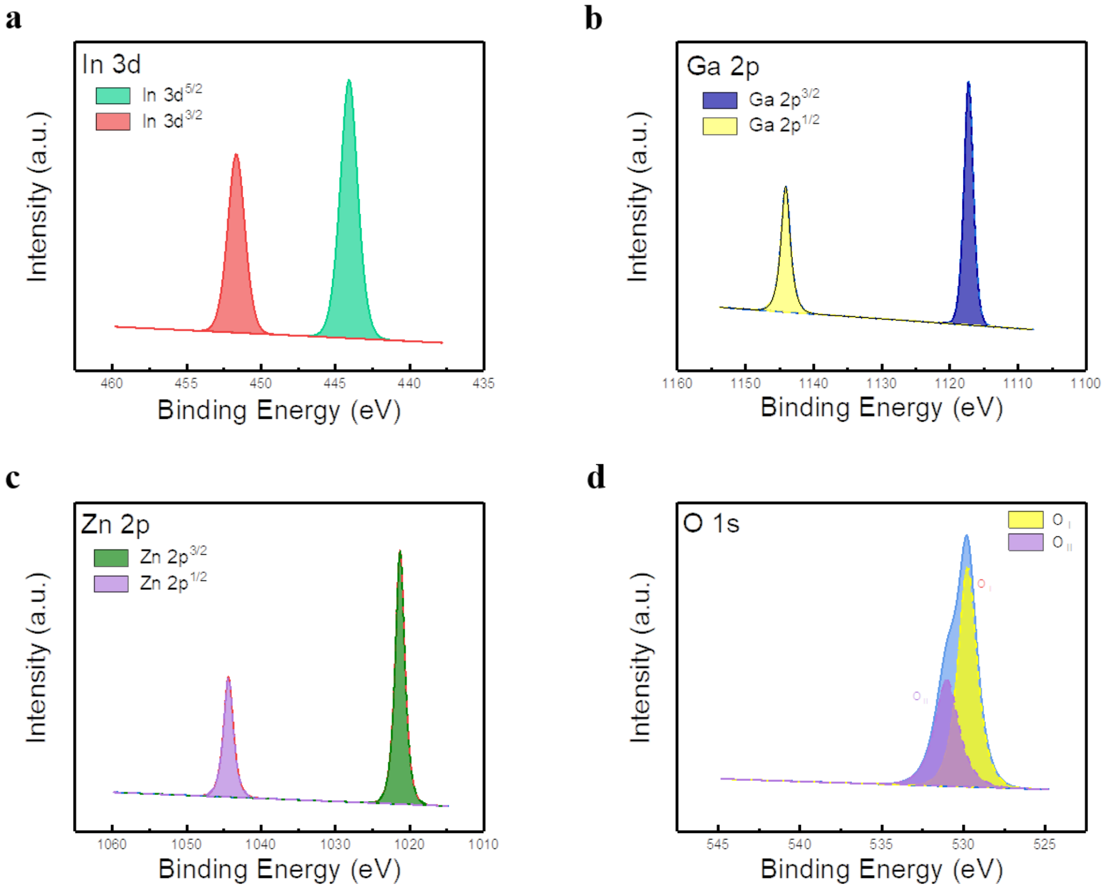

Figure S4. XPS spectra of the a-IGZO (a) In 3d (b) Ga 2p (c) Zn 2p (d) O 1s

At the In 3d peak, the binding energy for In $3d^{5/2}$ and In $3d^{3/2}$ were observed at 444.1 and 452 eV, respectively. These are attributed to the In^{3+} binding state in IGZO. For the Ga p2 peak, the binding energy for Ga $2p^{3/2}$ and Ga $2p^{1/2}$ were found to be 1117.3 and 1144.1 eV, respectively, indicating Ga^{3+} binding states in IGZO, In the Zn 2p peak, the binding energy for Zn $2p^{3/2}$ and Zn $2p^{1/2}$ were determined as 1021.3 and 1044.4 eV, respectively, suggesting Zn^{2+} binding states in IGZO. The O 1s peak of IGZO separates into two peaks at 529.8 and 531 eV. The lower binding energy peak (529.8 eV) corresponds to the binding energy of lattice oxygen (metai-oxide, O_I) while the peak at 531 eV is associated with non-lattice oxygen (oxygen vacancy, O_{II}). The areas obtained from the deconvolution of the O 1s peak (A_{OI} and A_{OII}) are utilized for the quantitative analysis of oxygen vacancies in the IGZO film using the following formulae:

$$O_I\,(\%) = \frac{AO\,\mathrm{I}}{AO\,\mathrm{I} + O\,\mathrm{II}} * 100 \qquad \text{(S1)}$$

$$O_{II}\,(\%) = \frac{AO\,\mathrm{II}}{AO\,\mathrm{I} + O\,\mathrm{II}} * 100 \qquad \text{(S2)}$$

Equations (S1) and (S2) are used to calculate the O_I and O_{II} area ratio of IGZO. The extracted IGZO O_I and O_{II} area ratios are 60.90 % and 39.10 %, respectively.

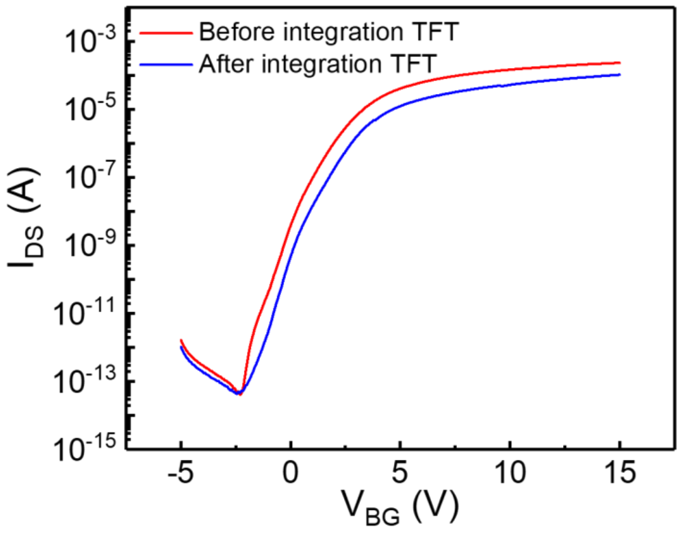

Device	I_{on}/I_{off}	μ_{FE}
Before integration TFT	$<10^9$	2.7635
After integration TFT	$<10^8$	2.2384

Figure S5. Comparison of Transfer Characteristics before and after Integration of a-Si:H Photodiode with a-IGZO TFT.

a b

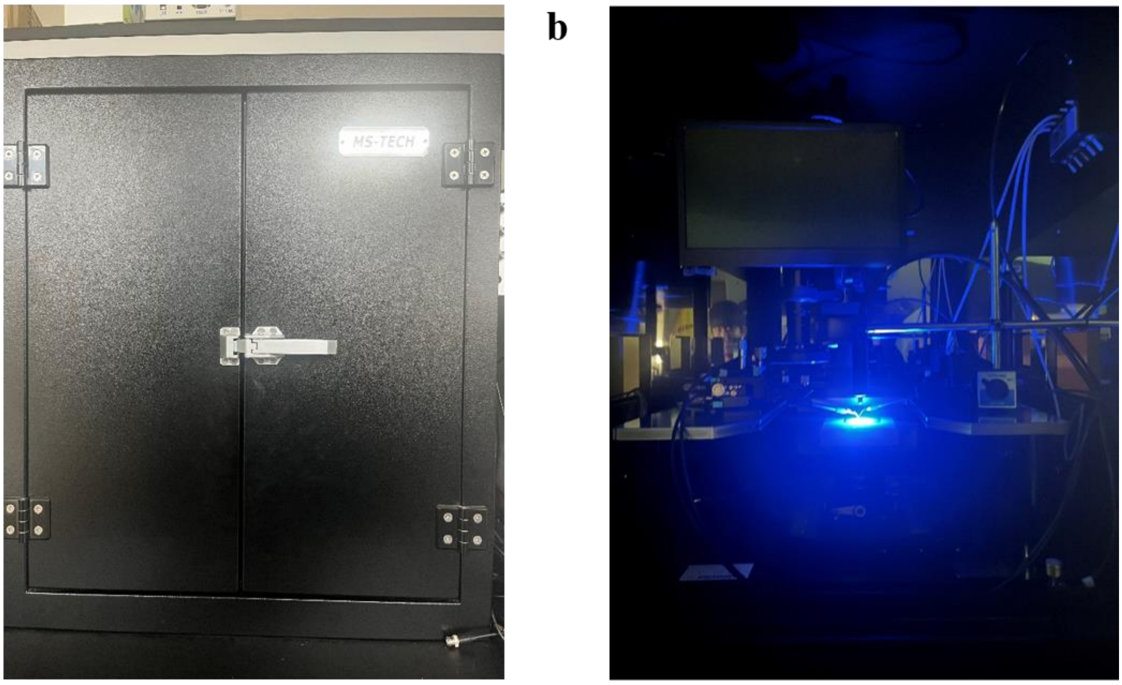

Figure S6. (a) Image of during the measurement of Dark current within the Dark box (b) Image of while measuring Photo current

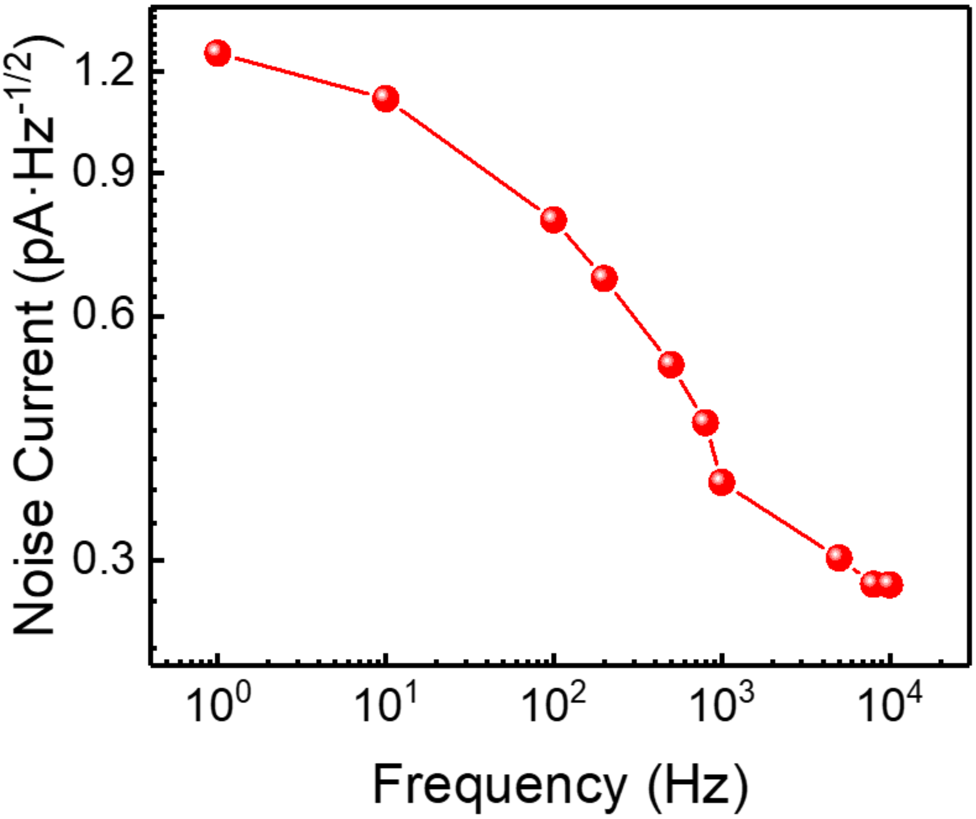

Figure S7. The noise current measurements of an integrated image sensor with a-IGZO tft and a-Si:H photodiodes in across a frequency range from 1 Hz to 10 kHz.

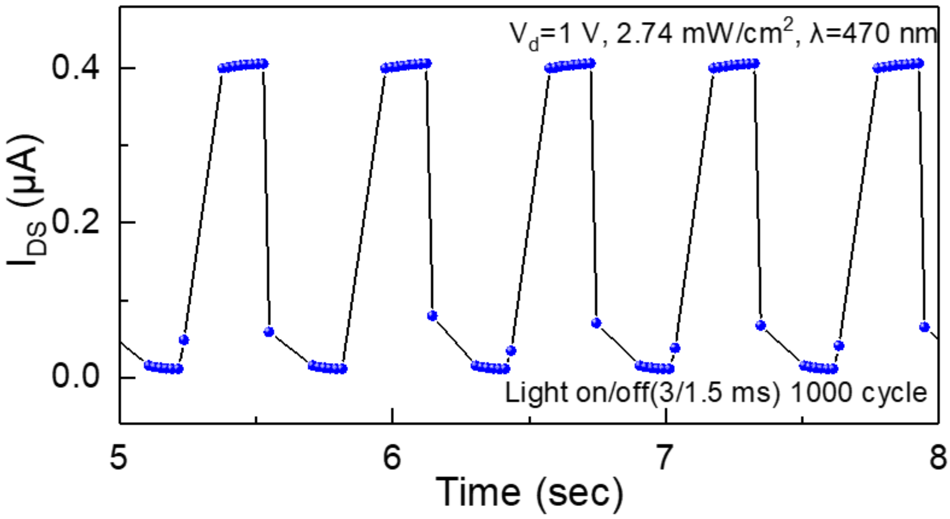

Figure S8. The rise time (140 ms) and fall time (21 ms) exhibited during the modulation of the light pulse on/off

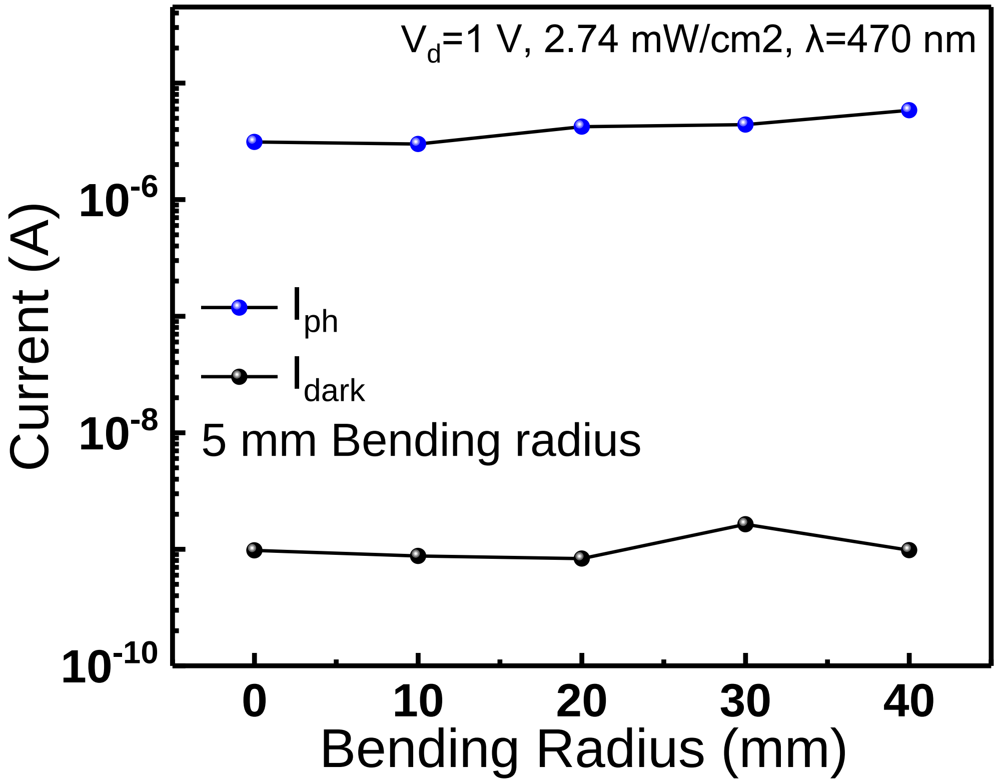

Figure S9. The variation in current with respect to bending radius when subjected to a light intensity of 2.74 mW/cm2 at a wavelength of 470 nm

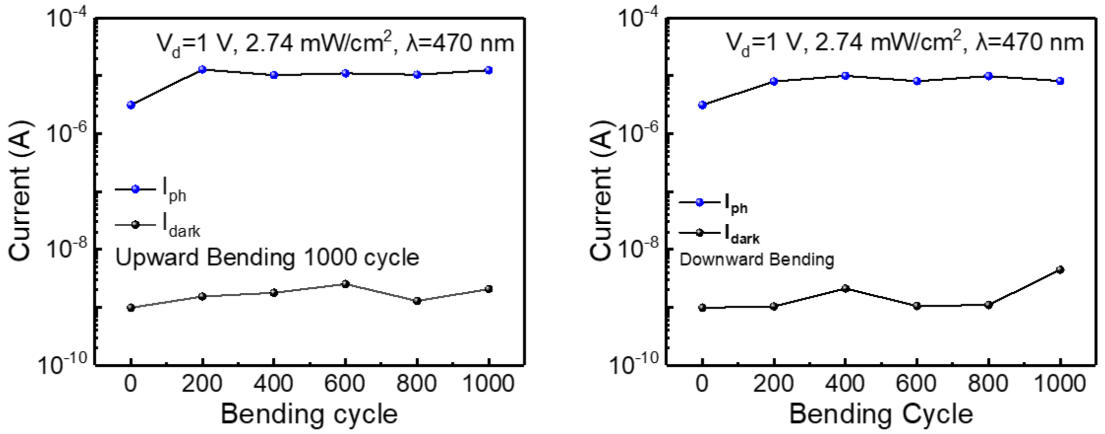

Figure S10. The current variation of J-V characteristics in response to bending cycles when exposed to upward bending and downward bending conditions under a light intensity of 2.74 mW/cm^2 at a wavelength of 470 nm.
